# Les tumeurs malignes naso-sinusiennes: à propos de 32 cas et revues de la littérature

**DOI:** 10.11604/pamj.2015.22.342.8220

**Published:** 2015-12-10

**Authors:** Youssef Darouassi, Mohamed Mliha Touati, Mehdi Chihani, Jihane El Alami, Brahim Bouaity, Haddou Ammar

**Affiliations:** 1 Service d’Oto-rhino-laryngologie, Hôpital Militaire Avicenne, Marrakech, Maroc

**Keywords:** Carcinome naso-sinusien, endoscopie endonasale, traitement, pronostic, nasalsinusal carcinoma, endonasal endoscopy, treatment, prognosis

## Abstract

Sous l’appellation tumeurs malignes naso-sinusiennes est regroupé un vaste éventail de tumeurs, aux histologies et localisations variées, mais aux tableaux cliniques souvent similaires. Le diagnostic de ces tumeurs est difficile, nécessitant une approche multidisciplinaire, à savoir oto-rhino-laryngologique, radiologique et anatomopathologique. Notre étude rétrospective concerne 32 cas de tumeurs malignes naso-sinusiennes, colligées au service d’ORL de l’hôpital militaire Avicenne de Marrakech, entre Janvier 2004 et Décembre 2014. L’analyse des données a noté que la fréquence des tumeurs épithéliales (75% des cas) était supérieure à celle des tumeurs non épithéliales (25% des cas), avec en tête de file l’adénocarcinome de l’ethmoïde (31,25%) et le carcinome épidermoïde du sinus maxillaire (18,75%). Ces tumeurs surviennent le plus souvent chez le sujet âgé avec une moyenne d’âge de 52 ans et une répartition équitable entre les deux sexes. Le délai de consultation moyen était de 12 mois avec une symptomatologie dominée par un syndrome nasosinusien (71,8%), associé dans certains cas à des signes ophtalmologiques (12,5%) ou neurologiques (15,6%). Tous nos patients ont bénéficié d’un examen clinique notamment endoscopique, d’une exploration radiologique des tumeurs et de leurs extensions, et d’une confirmation diagnostique par un examen anatomopathologique. Le traitement a consisté en une exérèse chirurgicale de la tumeur dès que cela était possible, soit dans 81,3% des cas (26 patients), généralement complété par un traitement adjuvant radio-chimiothérapique (77%). Le suivi à un an de nos patients a permis de noter une bonne évolution pour 08 d’entre eux (25%), une récidive dans 6 cas (18,75%), le décès de neuf patients (28,1%), et l’absence d’information concernant les autres cas (28,1%).

## Introduction

Les tumeurs malignes naso-sinusiennes représentent 3% des cancers des voies aérodigestives supérieurs (VADS) [1]. Développées dans des régions anatomiques confinées, et se manifestant par des signes cliniques anodins, elles amènent souvent à être traitées à des stades évolués et confrontent à des difficultés diagnostiques, thérapeutiques et psychologiques pour le patient.

## Méthodes

Il s’agit d’une étude rétrospective étalée sur 10 ans; de 2004 à 2014, portant sur 32 patients colligés au service d’oto-rhino-laryngologie (ORL) de l’hôpital militaire Avicenne de Marrakech, pour tumeurs malignes naso-sinusiennes (TMNS). Les cas inclus dans notre étude sont des patients, hommes et femmes de tout âge vus et traités au service pour TMNS, après confirmation anatomopathologique. Les cas exclus sont ceux dont l’examen anatomopathologique n’était pas concluant et ceux dont les dossiers étaient inexploitables. Une fiche d’exploitation comprenant les différentes données nécessaires à notre étude a été réalisée et remplie en ayant recours aux dossiers des patients. Cette étude a cherché à déterminer les aspects épidémiologiques, les manifestations radio-cliniques, le traitement prodigué ainsi que l’évolution et les complications survenues pour chaque patient.

## Résultats

**Figure 1:**
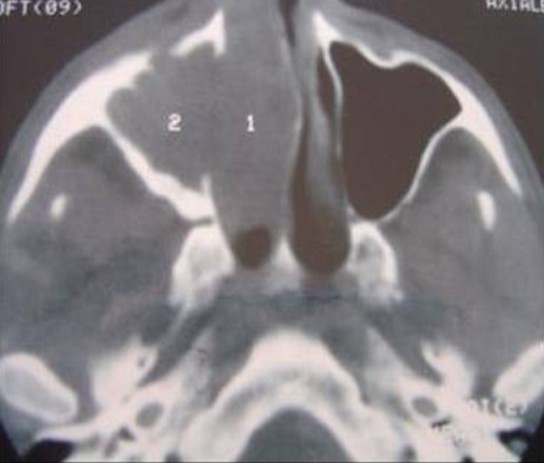
TDM naso-sinusienne en coupe axiale montrant un processus tumoral tissulaire occupant le sinus maxillaire droit lysant la paroi antérolatérale du sinus et repoussant la cloison nasale à gauche

La fréquence des tumeurs naso-sinusiennes est estimée à environ 3 cas/an dans notre étude. L’âge moyen de nos patients était de 52 ans. On note une répartition égale entre les 2 sexes. Les patients étaient essentiellement des militaires et leurs ayants droit. Un seul de nos patients était professionnellement exposé au bois. Le mode de début était progressif chez tous les patients avec un délai de consultation de 12 mois en moyenne. L’examen clinique et la symptomatologie étaient dominés par un syndrome nasosinusien (71,8%), associé dans certains cas à des signes ophtalmologiques (12,5%) ou neurologiques (15,6%). Le siège de l’atteinte était unilatéral chez tous les patients sauf un. L’endoscopie naso-sinusienne a permis de visualiser la lésion et de pratiquer des biopsies pour une analyse microscopique chez tous les patients. L’examen des aires ganglionnaires a permis de retrouver des adénopathies cervicales homolatérales chez quatre patients (au niveau de la région II dans deux cas, la région I dans un cas et la région III dans un cas). L’aspect radiologique habituel était une masse tumorale pleine, homogène, se rehaussant après injection d’iode, classiquement ostéolytique (82%) (Figure 1). Les calcifications intra-tumorales étaient également présentes (12,5%). La localisation initiale de la tumeur était ethmoïdale dans 40,6% des cas, maxillaire dans 28% des cas, nasale dans 12,5% des cas, sphénoïdale dans 6,25% des cas et enfin elle était d’origine indéterminée dans 12,5% des cas. Les différents types histologiques se répartissaient en tumeurs épithéliales (75%) et en tumeurs non épithéliales (25%) (Tableau 1).

**Tableau 1:** Répartition des tumeurs en fonction de leur type histologique

Types de tumeurs	Pourcentages	
Tumeurs épithéliales	Adénocarcinomes	37,5%
	Carcinomes épidermoïdes	21,9%
	Esthésioneuroblastome	9,4%
	Mélanome malin	3,1%
	Carcinome muco-épidermoïde	3,1%
Tumeurs non épithéliales	Sarcomes	12,5%
	Lymphomes malins non Hodgkiniens	12,5%

**Figure 2:**
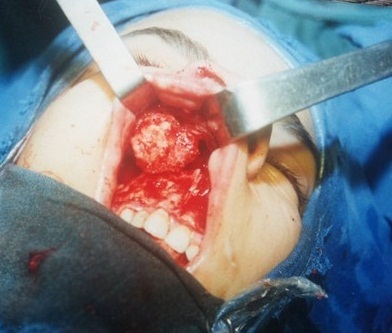
Incision vestibulaire droite pour exérèse chirurgicale d’un chondrosarcome maxillaire droit

Le bilan d’extension a permis d’objectiver un envahissement du cavum dans deux cas, un envahissement de la fosse infratemporale dans deux cas, un envahissement des tissus mous de la face en regard de la tumeur dans deux cas, des adénopathies cervicales dans 4 cas, et des métastases pulmonaires dans un cas. Parmi les patients, 83% présentaient un stade T3 ou T4 au moment du diagnostic. Concernant les lymphomes, il y avait trois patients en stades I (9,4%) et un en stade II (3,1%). Sur les 32 cas étudiés, 17 pont bénéficié d’un traitement chirurgical et d’une radiochimiothérapie post-opératoire, 9 patients ont subi un traitement chirurgical et une radiothérapie post-opératoire et 6 d’une radiochimiothérapie exclusive. Les reconstructions faciales ont été réalisées en peropératoire (Figure 2). Les suites post-opératoires étaient marquées par la survenue d’un cas de thrombose du sinus caverneux, d’une hypoesthésie de la deuxième branche du nerf trijumeau (V2), d’une méningite bactérienne, d’un cas de rhinorrhée cérébro-spinale, de trois cas de synéchies des fosses nasales, d’un diabète insipide, de deux épistaxis et d’une diplopie. Le reste des patients ont présenté des suites simples. Les suites lointaines, avec un recul d’un an, étaient marquées par une assez bonne évolution chez 08 patients, une récidive chez 06 patients, le décès de 09 patients et l’absence d’informations concernant les autres cas. Les patients qui nécessitaient une radiochimiothérapie ont été adressés au service d’oncologie de l’hôpital militaire Mohamed V de rabat pour prise en charge.

## Discussion

**Figure 3:**
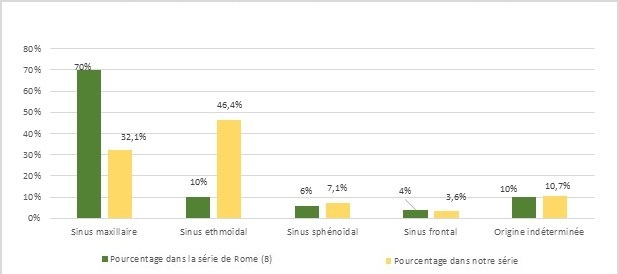
Répartition des tumeurs malignes sinusiennes en fonction de leur localisation initiale

L’incidence des tumeurs malignes naso-sinusiennes (TMNS) est relativement faible et correspond à 1/100000. Néanmoins, on observe un nombre plus élevé de femmes que dans les autres cancers des VADS avec un sex-ratio de 2:1 [1]. Dans notre série, le sex-ratio était de un. L’âge moyen de 52 ans dans notre série est inférieur à l’âge moyen décrit dans la littérature, avec une moyenne d’âge de 76 ans pour Garg et Mathur [2]. Seul un cas atteint d’adénocarcinome de l’ethmoïde était exposé au bois contre 80% dans la littérature [3]. D’autre part, 37,5% de nos patients, tous des hommes, étaient connus alcoolo-tabagique, facteur de risque pourtant peu décrit dans la littérature pour les cancers naso-sinusiens [4]. Le délai de consultation était souvent élevé, 12 mois dans notre série contre 4,4 mois dans la série de Boudet [4]. Ceci s’explique par l’apparente banalité des symptômes et la négligence de la part des patients. Cliniquement, les signes naso-sinusiens étaient les plus fréquents dans notre série (71,8%) conformément à ce qui est décrit dans la littérature. Une extension de la tumeur était responsable de signes extra naso-sinusiens, notamment ophtalmologiques (12,5%) et neurologiques dans 15,6% des cas, pourcentages similaires à la série de Boudet [4]. Les cancers naso-sinusiens sont peu lymphophiles. La littérature donne des chiffres allant de 6 à 21%, ce qui correspond au constat dans notre série où 04 cas d’envahissements ganglionnaires (12,5%) ont été notés. Dans la littérature, 20% de ces cancers se développent au niveau de l’ethmoïde, 70% au niveau du sinus maxillaire et 10% au niveau des fosses nasales [5]. Ses proportions étaient similaires dans la série de Rome [6] qui ne comprend que des tumeurs sinusiennes (Figure 3). Dans notre série, le point de départ des tumeurs était essentiellement ethmoïdal. La diversité de ces cancers est grande, néanmoins il a été trouvé que les tumeurs épithéliales étaient plus fréquentes (83,3%) que les tumeurs non épithéliales (16,6%) [5, 6]. Les carcinomes épidermoïdes sont généralement décrits comme étant les plus fréquents, comme en témoigne cette étude multicentrique réalisée en France (Tableau 2) sur 700 cas de tumeurs du massif facial supérieur [7].

**Tableau 2:** Comparaison du pourcentage des tumeurs malignes en fonction de leur nature histologique

Type histologique de la tumeur	Dans une série de 700 cas [7]	Dans notre série
Carcinomes épidermoïdes	48,5%	21,9%
Adénocarcinomes	22%	37,5%
Lymphomes malins non Hodgkiniens	11%	12,5%
Carcinomes adénoïdes kystiques	10,5%	0%
Carcinomes muco-épidermoïdes	0%	3,1%
Esthésioneuroblastomes	3%	9,4%
Sarcomes	3%	12,5%
Mélanomes malins	2%	3,1%

Dans notre série, le ratio carcinomes épidermoïdes / adénocarcinomes est inversé. Nous avons également retrouvé un carcinome muco-épidermoïde (3,1%) alors qu’au sein des tumeurs d’origine salivaires, le carcinome adénoïde kystique paraît plus fréquent [7]. 82% des patients étaient en stade T3 ou T4 lors de la première consultation, chiffres en accord avec la série de Boudet (76% des cas) [4]. Les facteurs suivants (Tableau 3) ont fréquemment été attribués à une augmentation du risque de cancer des fosses nasales et des sinus paranasaux [3, 8]. L’exploration des tumeurs du massif facial relève actuellement du scanner et de l’IRM [9]. Tous les patients de notre série ont bénéficié d’un bilan scannographique. Certains critères étaient considérés comme suspects: l’ostéolyse, le caractère invasif et dans une moindre mesure, l’unilatéralité de l’atteinte [4]. Néanmoins l’imagerie ne permet pas la différenciation entre bénignité et malignité. Cette difficulté est en général résolue, par l’accès à la biopsie directe de la plupart de ces tumeurs [9]. L’imagerie par résonance magnétique (IRM) est particulièrement utile car elle permet d’analyser de façon plus précise le volume et les extensions tumorales [9]. La chirurgie endoscopique représente l’étape la plus récente des techniques chirurgicales en rhinologie. Utilisée au début dans de rares cas de TMNS localisées, elle est actuellement de plus en plus employée, y compris dans certains cas évolués. La chirurgie par voie externe (voies paralatéronasale et sous-labiale) garde toutefois une grande place et a été pratiquée chez l’ensemble de nos patients. La radiothérapie constitue un des éléments essentiels du traitement des TMNS [7]. En effet tous nos patients en ont bénéficié en post-opératoire. La place de la chimiothérapie dans la stratégie thérapeutique est moins établie, exception faite des lymphomes naso-sinusiens [4] traités par radiochimiothérapie, comme cela a été le cas dans notre série. 18,75% des patients ont présenté une récidive dans l’année qui a suivie, chiffre conforme à la littérature [4]. La mortalité à un an était de 28,1%, proche de celle de Boudet (20%) [4]. Enfin, les progrès actuels dans la chirurgie robotisée donnera probablement de nouvelles perspectives dans les années à venir [10].

**Tableau 3:** Facteurs de risque connus et possibles des cancers naso-sinusiens

Facteurs de risque connus	Facteurs de risque possibles
- Poussières de bois	- Virus du papillome humain (VPH)
- Poussières de cuir	- Fumée secondaire
- Composés de nickel	- Formaldéhyde
- Virus d’Epstein-Barr	- Chrome
- Papillome inversé	- Poussière de tissu
	-Radiothérapie pour un rétinoblastome

## Conclusion

L’étude de cette série de cancers naso-sinusiens montre l’extrême gravité de ces tumeurs, dont le pronostic reste sombre, malgré la multiplication des moyens diagnostiques et thérapeutiques actuels. Il faut s’attacher à surveiller au mieux ces patients par des examens cliniques, endoscopiques, et radiologiques réguliers, pour dépister au plus tôt d’éventuelles récidives. Les professionnels exposés doivent faire l’objet d’une surveillance particulière et de mesures de préventions spécifiques. Le pronostic restant étroitement lié à un diagnostic précoce et au contrôle local de la maladie.
